# Nurse Registration in America

**Published:** 1918-07-13

**Authors:** Carrie M. Hall

**Affiliations:** Chief Nurse A.R.C. in Great Britain.


### THE MATRONS' AND SISTERS' DEPARTMENT.

### NURSE REGISTRATION IN AMERICA.

By MISS CARRIE M. HALL, R.N., A.N.C., Chief Nurse A.R.C. in Great Britain.

WHEN I came over last year, I came with a steamer trunk and camp kit to do war nursing in France. I left all the cares of the training-school and the problems of the nursing profession behind. I brought no books or other material with me from which to draw data. What I say, therefore, will have to be from memory. I venture to say that your President could tell you more about State Registration of nurses in America than I can. It is a matter of history that it was Mrs. Bedford Fenwick who suggested to the nurses of America in 1889 that it would be a good thing to have State Registration of nurses. The campaign was begun in 1901 by the forming of State Associations. These Associations at once took up the work of securing laws in their various States. In America each of the States has to enact its own laws, and I believe that a Nurses' Registration Law has been enacted in forty-six different States. No two of these laws are exactly alike, but in fundamental things they are much the same in all the States. The State law usually reads something like this:—" Any woman of twenty-one years or over, of good moral character, having graduated from a training-school for nurses giving a systematic course of instruction and attached to a hospital of a definite number of beds and with a stated average daily number of patients, and having undergone a course of instruction of at least two years in such hospital, may practise as a registered nurse, using the letters R.N. after her name." The size of the hospital and the average daily number of patients required as material for suitable training varies in the different States. The number of years demanded varies. Some States require three years of training. The standard of admission to a training-school varies from a complete High School course, or its equivalent, as a minimum standard, down to mere preparation for admission to a High School. In most States the law did not in the first instance make registration compulsory, but protected the title of registered nurse. In several States it has since been amended and registration is compulsory. All registered nurses are required to have general training, and, generally, each training-school makes its own affiliation with other hospitals to secure training for its pupils in those branches in which it lacks the necessary material. Nearly every Bill has provided a waiver, so-called, providing that those nurses who had graduated previous to the passing of the Bill, and those in training at the time of its passage, might be registered without examination. The period of the waiver is generally three years, at the end of which time no one could become a registered nurse without passing the State Board examination. The constitution of the Boards of Examiners varies greatly. Some consist of five members and others of seven. Some are wholly composed of medical men, others of two nurses and three medical men, others again of three nurses and two medical men, while in a few instances the Boards are made up entirely of nurses. We believe that expert nurses are better fitted to examine nurses and pass upon their qualifications than medical men. In nearly all States the Boards of Examiners are appointed by the Governor of the State, but almost invariably the nursing representatives are chosen from candidates selected or nominated by the State Association of Nurses. The passage of the registration laws has brought home forcibly the need of State inspection of training-schools. In a few States, provision was made for inspection with the passage of the original law. In other States, it has since been adopted. Others are still hoping to get it.

Nurses have had an answer to the question during the last year, if never before, how registration affects the individual nurse. The Red Cross organisation has always stood for high standards in nursing education, and it is a necessary qualification for enrolment as an American Red Cross nurse that the applicant must be a registered nurse. That, at any rate, was the rule up to six months ago, and hundreds of nurses who could not become enrolled as Red Cross nurses, because they were not registered, began to look around and see what registration was, anyway. Probably the most noticeable result of registration has been the need for more legislation. It has constantly been necessary to improve and amend the laws. The preliminary work, including the forming of the Bills and getting them before the different Legislatures in proper form, has perhaps made American nurses something of politicians, and certainly a large majority of them are now ardent Suffragists.

